# 

**Published:** 1886-04

**Authors:** 


CONTENTS

Vol. 33 No. 4

| | PAGE. |
|---|---|
| Natural Healing | 97 |
| Lying With the Head High | 99 |
| Deadly Drugs and Curious Antidotes | 100 |
| Mouth Breathing | 102 |
| An Important Admission | 105 |
| To a Correspondent | 106 |
| Nerves and Irregular Hours of Sleep | 107 |
| The Human Ra e | 108 |
| St. Vitus Dance | 109 |
| Disinfectants | 110 |
| Sight | 110 |
| The Dietary in Indigestion | 112 |
| Miscellaneous Items | 116 |
| D. F. Wright, M. D., on Eggs | 120 |
| Wall Paper Peril | 124 |
| Health Alphabet | 124 |
| Who Use Bread | 125 |
| Home Department | 126 |
| Moller's Norwegian Cod Liver Oil | 128 |
| Whooping Cough Cured | 128 |

